# Crash severity analysis of nighttime and daytime highway work zone crashes

**DOI:** 10.1371/journal.pone.0221128

**Published:** 2019-08-13

**Authors:** Kairan Zhang, Mohamed Hassan

**Affiliations:** 1 National Engineering Laboratory of Integrated Transportation Big Data Application Technology, Southwest Jiaotong University, Chengdu, Sichuan, China; 2 National United Engineering Laboratory of Integrated and Intelligent Transportation, Southwest Jiaotong University, Chengdu, Sichuan, China; University of British Columbia, CANADA

## Abstract

**Introduction:**

Egypt’s National Road Project is a large infrastructure project which presently aims to upgrade 2500 kilometers of road networks as well as construct 4000 kilometers of new roads to meet today’s need. This leads to an increase in the number of work zones on highways and therefore a rise in hazardous traffic conditions. This is why highways agencies are shifting towards night construction in order to reduce the adverse traffic impacts on the public. Although many studies have investigated work zone crashes, only a few studies provide comparative analysis of the difference between nighttime and daytime work zone crashes.

**Methods:**

Data from Egyptian long-term highway work zone projects between 2010 and 2016 are studied with respect to the difference in injury severity between nighttime and daytime crashes by using separate mixed logit models.

**Results:**

The results indicate that significant differences exist between factors contributing to injury severity. Four variables are found significant only in the nighttime model and four other variables significant in the daytime model. The results show that older and male drivers, the number of lane closures, sidewise crashes, and rainy weather have opposite effects on injury severity in nighttime and daytime crashes. The findings presented in this paper could serve as an aid for transportation agencies in development of efficient measures to improve safety in work zones.

## Introduction

The number of road fatalities is a critical issue in Egypt. In 2013 alone, 10,466 were killed in road crashes. Among the casualties, young and middle-aged people suffer the highest number of fatalities. This has a major impact on the society and the economy [1,2].

In the National Roads Project, more than 4000 kilometers of new roads are to be constructed to strengthen the Egyptian road network, with another 2500 road kilometers being upgraded [3], leading to a large increase in the number of work zones. The work zones interfere with traffic due to the conflicting needs between traffic flow and construction and maintenance activities. Closing highways for traffic entirely during construction and maintenance work is usually unrealistic. Many highway planning and construction agencies are consequently shifting to nighttime work activities to lessen the impact on travelers (lower congestion levels), increasing productivity due to less interference and quicker material delivery (less machinery idle time), creating a safer work environment and taking advantage of cooler working conditions at nighttime. According to a comprehensive survey of work zone incidents in the USA, 58% of vehicle crashes occurred at daytime work zones, 33% at nighttime sites, and 9% at zones with construction work ongoing both day and night [4]. Research on road work zone safety conditions at day and nighttime construction sites by Arditi et al. revealed that work zone crashes during the night tend to be more severe than at daytime [5]. A number of studies supported the conclusion that collisions usually are more severe at nighttime than during the day [6–9]. In contrast, an investigation by Garber and Zhao concluded that there were no large difference in work zone crash severity between day and nighttime [10].

Most crash studies do not distinguish between incidents occurring in daylight and at nighttime. This is partly due to lack of detailed data, partly on the assumption that the same conditions prevail at all times of the day. However, driving conditions differ significantly between day and night by changing environment, traffic pattern as well as driver behavior, all influencing the crash likelihood. For example, as the temperature tends to drop at night, roads are more likely to become slippery than during the day. In addition, human factors like fatigue, drinking-and-driving and speeding are more prevalent in the nighttime.

The increasing presence of work zones and the difference in driving conditions between daytime and nighttime provides a strong motivation for further investigation of work zone crashes. The severity of injury following collisions is of primary interest to researchers in traffic safety, aiming at designing measures both to prevent crashes and decreasing their severity [11–13]. An important step in this research is the identification of significant factors influencing injury severity.

## Literature review

Traffic safety has long been recognized as an important issue due to the large economic and social impacts of road crashes. Injury severity following crashes is a parameter of primary interest to researchers since it directly reflects social and economic damage and an understanding of how it is influenced by independent factors aids in designing measures preventing crash incidents as well as mitigating their severity. Researchers have applied various statistical models to identify the factors affecting injury severity. For instance, Guo et al. investigated the impact of various risk factors on three types of collisions at freeway diverge areas and utilized the random parameters multivariate Poisson-lognormal model [14]. The random parameters multivariate Tobit model was used by Guo et al. to evaluate the risk factors on crash rates of different collision types [15]. On the same note, Guo and Sayed a study using Four candidate Tobit models to evaluate the impact of various risk factors on crash rates at freeway diverge areas [16]. By using random parameters multinomial logit regression, Guo et al identified significant factors contributing to the severity of e-bike collisions [17].

The light conditions while driving have long been considered a significant parameter for the frequency and severity of work zone crashes. A thorough understanding of this relationship is fundamental for transportation agencies when selecting appropriate work zone strategies.

Many researchers have found an increase in both crash frequency and severity in the nighttime [8,18–22]. For instance, Johansson et al. reported an increase in crash injury risk at night in urban, rural, and both area types together of 30%, 50% and 40%, respectively. Abdel-Aty showed that crashes occurring during adverse weather and poor light conditions were significantly associated with higher injury severity [6]. Sze and Soong examined the relationship between work zone crash severity and common influential factors in New Zealand using a multinomial logistic regression model. The study concluded that crashes in the daytime were associated with more severe injuries [23]. In contrast, the work by Osman et al. concluded that work zone crashes tended to lead to higher severity during the evening and late night [24]. An analysis of crash records from Alabama work zones revealed that lack of lighting contributed to the frequency and severity of such crashes, emphasizing the importance of adequate lighting at work zones during the night [25].

By applying the ordered probit model, Jafari and Hadji conducted a study to examine the effects of lighting conditions on the severity of crashes occurring on two-lane rural roads. The researchers concluded that increased daylight crash risk was associated with higher speed limits and curved sections, while rear-end crash type was associated with increased nighttime crash risk. In addition, the researchers suggested that intersection lighting on two-lane rural roads could reduce crash injury severity [26]. With a similar approach, Dias and Dissanayake used the ordered probit model to identify factors contributing to increased injury severity of nighttime and daytime work zone collisions using crash data from 2010–2013. The crash location was found to be significantly associated with the severity of the crash only during daytime and likewise, work zone location was found to be significantly associated with crash severity only during nighttime [27]. To determine the significant contributing factors for work zone crashes under different light conditions, Wei et al. developed a classification and regression tree model showing that nighttime driving at high speeds under the influence of drugs or alcohol in poorly lit areas lead to an increase the severity level [28].

In analysis of crash data, the multinomial logit (MNL) and its generalizations are one of the most commonly deployed regression models for representing graded crash severity outcomes [29]. The MNL has been used to examine the injury severity of crashes involving trucks [30], motorcycle crashes [31], and crossover and rollover crashes [32]. The MNL has also been used to model severity of vehicle collisions involving pedestrians [33], farm vehicle crashes [34] and highway work zones in New Zealand [23]. In a study of factors influencing crash severity in collision data in Germany, Manner and Wünsch-Ziegler used MNL models to predict injury severity levels from independent factors and concluded that night time is the most influential factor for higher crash severity level [35]. By using MNL, Guo et al examined the factors that influences the registration of electric bikes [36]

The mixed multinomial logit (MMNL) model is a generalization of the traditional multinomial logit model allowing random regression coefficients thereby capturing effects caused by variations in unobserved variables. The random errors are assumed to be distributed according to a type I extreme value distribution. Predictions based on MMNL regression can therefore be expected to be more accurate than the results from the standard MNL model because it is a more flexible and powerful approach relaxing the limitations of a MNL model [17,37–41].

In Egypt, there is a lack of published data that pertains to day and nighttime crashes and how they relate to work zones. In addition, research results based on data from western countries may not be directly applicable to developing countries such as Egypt due to differences in roadway designs, traffic characteristics and driver behavior. The present study is therefore an attempt to bridge this knowledge gap. The MMNL model is used on Egyptian data to identify explanatory factors which are significant on injury severity levels of daytime and nighttime work zone crashes, and to find the strength of correlation between these factors and the outcome.

## Materials and methods

### Dataset characterization

This study is an investigation into work zone crashes in Egypt that occurred during ten long-term highway maintenance and rehabilitation projects (having a duration longer than one year) in the period 2010–2016, with the aim at determining significant factors influencing severe injury resulting from daytime and nighttime work zone crashes analyzed separately. The crashes are categorized according to the time of the crash. Daytime is defined as the time between 6:00 a.m. to 5:59 p.m., and nighttime between 6:00 p.m. and 5:59 a.m. A total of 1,380 incidents were collected in the time period, among which 746 crashes occurred during nighttime and the remaining 634 crashes occurred during the day. Additional details recorded for each incident included information about the driver and the vehicle, and characteristics of the road, the work zone and the environment.

In this paper, the severity of a crash is determined based on the single most serious injury suffered. In case of at least one fatality, the incident is classified as fatal (labeled ‘fatality’), and when at least one injury is sustained, it is classified as injurious (labeled ‘injury’). Crashes that do not result in injuries are categorized and labeled as ‘PDO’.

Closing off highways to traffic, while maintenance and rehabilitation work is ongoing is very difficult. Sometimes half of the road has to be open to traffic during work on the other half. Since this situation is inevitable, this paper has taken into account the types of surface construction to uncover which surface conditions contribute to daytime and nighttime crashes in work zones. In this regard, the type of surface construction for each crash is divided into five categories reflecting the condition of highway surfaces (Asphalt, Milling, Concert, Removing asphalt and Base). Table 1 shows the descriptive statistics and frequency distribution of the factors considered in the analysis.

**Table 1:** Descriptive statistics of key variables in the models.

Variable	Daytime crashes		Nighttime crashes	
	Proportion%	S.D	Proportion%	S.D
**Driver characteristics**				
Young driver is under <35 (1 if true; 0 otherwise)	60.7	0.481	74	0.439
Middle driver is between 35–50 (1 if true; 0 otherwise)	30.1	0.459	22.9	0.421
Old driver is above > 50 (1 if true; 0 otherwise)	9.2	0.240	3.1	0.173
Male driver (1 if true; 0 otherwise)	84.4	0.363	92	0.272
**Roadway characteristics**				
Curve (1 if crash occurred in curve section; 0 otherwise)	12.0	0.325	14.1	0.348
Straight section (1 if true; 0 otherwise)	45.5	0.496	43.4	0.496
U-turn section (1 if true; 0 otherwise)	23.5	0.388	14.7	0.355
Straight & grade road (1 if true; 0 otherwise)	19.0	0.439	27.7	0.448
Rural area (1 if true; 0 otherwise)	61.3	0.374	72.8	0.445
**Crash characteristics**				
Multi-vehicle crash (1 if true; 0 otherwise)	82.5	0.380	79.5	0.404
Heavy vehicle (1 if true; 0 otherwise)	29.2	0.408	35.9	0.48
Passenger vehicle	73.7	0.522	61	0.488
Angle-side collision	21.1	0.409	22	0.414
Sideswipe collision	32.9	0.449	24.4	
Rear-end collision	27.4	0.475	33.9	0.474
Fixed object collision	11.0	0.325	16.6	0.373
Pedestrian collision	7.6	0.209	3.1	0.173
**Environmental characteristics**				
Weekday (1 if crash occurred on a weekday; 0 otherwise)	74.2	0.335	67.6	0.468
Winter season (Dec.–Jan.–Feb.)	37.9	0.485	41.4	0.493
Summer season (Jun.–Jul.–Aug.)	11.0	0.314	19.6	0.397
Clear weather condition (1 if true; 0 otherwise)	53.2	0.499	57.5	0.495
Foggy weather condition (1 if true; 0 otherwise)	33.0	0.470	25.6	0.437
Rainy weather condition (1 if true; 0 otherwise)	13.9	0.346	16.9	0.375
**Work-Zone information**				
Asphalt (1 if surface construction is asphalt; 0 otherwise)	22.7	0.419	20.4	0.403
Base (1 if construction is base; 0 otherwise)	17.7	0.382	15.4	0.361
Remove asphalt construction (1 if true; 0 otherwise)	26.2	0.440	31.4	0.464
Milling construction(1 if true; 0 otherwise)	6.9	0.254	18.1	0.385
Concrete construction (1 if true; 0 otherwise)	26.5	0.442	14.7	0.355
More than one lane closures (1 if true; 0 otherwise)	41.3	0.435	58.4	0.493
Exceeded posted speed limit (1 if true; 0 otherwise)	57.9	0.464	62.1	0.486
**Crash severity**				
Property Damage Only (PDO)	24.0%		18.4%	
Injury	56.5%		53.7%	
Fatal injury	19.5%		27.9%	

### Methods

In order to study the work zone crash data, the general regression model

$$y_{j}=x_{ij}∙\beta_{i}+\epsilon_{j}$$ (1A)

$$\beta_{i}∼g(\beta |\theta )$$ (1B)

is used, where *i*∈*I* is an index set representing explanatory category variables, *j* = 1,2,…,*n* the index of observations, *y*_*j*_ the dependent variable for observation *j*, *β*_*i*_ a vector of random regression coefficients, *x*_*ij*_ a vector of observed explanatory variables, and *ϵ*_*j*_ an error term of observation *j*, assumed to follow an extreme value distribution type I. For the standard multinomial logit model, the outcome probability densities for the MNL can be written

$$p_{j}(i=k)=\frac{\exp\left(\beta_{k}x_{kj}\right)}{\sum_{I}\exp\left(\beta_{i}x_{ij}\right)}$$ (2)

where *β*_*i*_ are determined by maximum likelihood estimation. In the mixed multinomial logit model, the regression parameters *β*_*i*_ are allowed to be random variables across the set of observations with probability density function *g*(*β*|*θ*) with expectation *β*. In the MMNL model, the probability of an observation *j* having an outcome *k* is given by

$$p_{j}(i=k)=\frac{\exp\left(\beta_{k}x_{kj}\right)}{\sum_{I}\exp\left(\beta_{i}x_{ij}\right)}g(\beta |\theta )d\beta$$ (3)

The probability density of the parameters is often assumed to be the multivariate normal distribution, that is

$$\beta_{i}∼g(\beta |\theta )=N(\bar{\beta_{i}},\sigma_{i}^{2})$$ (4)

Where $\bar{\beta_{i}}$ is the vector of the mean values of the regression coefficients. When the variance $\sigma_{k}^{2}=0$ for all *i* the model reduces to the fixed-parameter MNL case. To determine the random regression parameters it is typically necessary to resort to simulated (Bayesian) maximum likelihood estimation (SML). Since for any *i* = *k*. *β*_*k*_ is not observable, the parameter can be integrated out from the conditional distribution *g*(*β*|*θ*) to obtain

$$P_{k}(\theta )=\int_{\beta_{k}}P(y_{k}|x_{k},\beta_{k})g(\beta_{k}|\theta )d\beta_{k}$$ (5)

That is, the cumulative probability function corresponding to (3). This equation has no closed-form solution, so it is solved by Monte Carlo integration, yielding an approximation *P*_*k*_′(*θ*) of the factor in the maximum likelihood function. For any given values in the parameter vector *θ*, a value of the parameter vector *β*_*kr*_ is sampled in a random draw *r*, from which $P_{k}^{\prime }(y_{k}|x_{k},\beta_{k})$ is calculated from (3) and (4). The estimated value *P*_*k*_′(*θ*) of *P*_*k*_(*θ*) is the average value of a total of *R* draws.

It can be shown that the SML estimator is consistent and asymptotically normal under some regularity conditions. The SML usually requires a large number of samples to give estimates of sufficient precision, which can be very time consuming. In order to keep the number of draws reasonably low, the points are drawn from a Halton sequence, which has better coverage than pseudo-random number generators. A typical choice of sample size is *R* = 200.

In addition to the regression parameters, the direct pseudo-elasticity of the independent variables on the outcome is defined as the relative change in probability after a unit change (1%) in the independent variable. Formally, the pseudo-elasticity is given by

$$E_{x_{jk}}^{p_{j}(i)}=\exp\left(\beta_{ik}\right)\frac{\sum_{k\in I}\exp\left(\beta_{k}x_{kj}{|}_{x_{jk}=0}\right)}{\sum_{k\in I}\exp\left(\beta_{l}x_{ij}{|}_{x_{jk}=1}\right)}-1$$ (6)

From this expression, the sensitivity of the ordered outcomes on the independent variables can be assessed. In this study, the statistical software R, is used for model parameter estimation.

## Results and discussion

### Model estimation results

The data is divided into two parts, corresponding to daytime and nighttime crashes, respectively. For the purpose of the analysis, the injury severity of work zone crashes is modeled by an MMNL model and an MNL model for comparison. The severity is divided into three categories. For brevity, we abbreviate the injury severity categories as ‘PDO’ for ‘Property Damage Only’, ‘IN’ for injury and ‘F’ for ‘fatality’. The ‘PDO’ category is chosen as the base case so that the estimated parameters in Table 2 and Table 3 show the difference between the results of the target category and the base case. The first step in the modeling process is testing for multicollinearity of the variables and those passing the test are entered into the model. The data multicollinearity is tested by calculating the variance inflation factor (VIF) for each explanatory variable, which indicates how much the variance of an estimated regression coefficient increases if the predictors are correlated. The explanatory variables chosen to enter the model are those with VIF values lower than 5; variables with higher values are excluded to avoid problems arising from multicollinearity. The stepwise elimination procedure is carried out to select the significant variables for the final model. The McFadden pseudo−*R*^2^ of the nighttime and daytime models equals 0.37 and 0.49 respectively, which indicate that the models fit the data satisfactorily. Two variables from the models have statistical significance at confidence interval of 90%, while other variables were significant at 95% confidence level.

**Table 2:** Parameter estimates for nighttime injury severity MMNL model.

Variable	Coefficient (standard deviation)	t-Statistic
Constant [IN]	2.065	2.788
Constant [F]	4.444	3.85
Young [IN]	-1.508	-3.336
Young [F]	-0.987	-1.988
Old [IN]	-2.441	-2.716
Old [F]	-2.955	-2.417
Male [IN]	1.460	2.827
Male [F]	2.060	3.601
Curve [F]	1.755	2.486
U-turn [IN]	3.135	3.37
Rural [IN]	1.001	2.114
Rural [F]	1.169	2.369
Angle crash [F]*	-1.324	-1.745
Sideswipe [IN]	-4.279	-4.213
Side swipe [F]	-2.405	-2.831
Heavy [IN]	1.145	2.832
Heavy [F]	2.218	5.181
Passenger [IN]	5.380	9.579
Passenger [F]	6.335	10.098
Multi-vehicle [F]	-1.98	-3.087
Winter [IN]*	0.603	1.889
Winter [F]	0.833	2.407
Rain [IN]	2.199	4.083
Concrete [IN]	1.341	2.422
Asphalt [IN]	0.986	1.984
Milling [IN]	-1.565	-3.508
Milling [F]	-2.557	-5.053
N. lane closures [F]	0.971	2.198
Speeding [IN]	1.26 (0.467)	2.706
Speeding [F]	1.402	2.796
N observations		746
Log-likelihood at convergence		-470.05
McFadden pseudo R^2^		0.371

Note: Variables (*) are significant at 90% confidence level.

**Table 3:** Parameter estimates for daytime injury severity MMNL model.

Variable	Coefficient	t-Statistic
Constant [IN]	2.583	1.884
Constant [F]	0.579	.344
Young [F]	2.073	3.756
Old [F]	2.922	2.043
Male [F]	-1.235	-2.169
Curve [IN]	-1.729	-2.639
Curve [F]	4.352	4.274
Rural [IN]	1.773	4.504
Rural [F]	1.887	3.555
Side swipe [F]	-2.523	-2.918
Pedestrian [IN]	2.732	2.182
Pedestrian [F]	3.763	3.823
Heavy Vehicle [F]	1.972	4.353
Passenger [IN]	3.530	8.498
Passenger [F]	11.316	7.329
Multi-vehicle [IN]	-2.969	-4.162
Multi-vehicle [F]	-6.047	-5.056
Weekday [F]	-1.870	-2.121
Rain [F]	-4.958	-4.120
Fog [IN]	-1.039	-2.735
Fog [F]	-2.936	-4.666
Concrete [F]	-2.980	-3.879
Asphalt [IN]	1.299	2.515
Remove Asphalt [F]	-3.114	-1.772
Milling [F]	-6.382	-5.646
N. lane closures [F]	-1.722	-2.799
N observations		634
Log-likelihood at convergence		-314.89
McFadden pseudo R^2^		0.495

In the nighttime model, the only remaining random variable is speeding, which is modeled as normally distributed with mean 1.263 and standard deviation 0.967. For this variables 9.6% of the distribution is less than 0 and 90.4% is greater than 0. This indicates that 9.6% of the high-speed vehicles involved in nighttime work zone crashes result in a decrease in injury level, while 90.4% of the crashes result in an increase in injury level. In the daytime model, no parameter is found significant when randomized, so the parameters become fixed.

The MMNL model parameters for the nighttime and daytime models are presented in Table 2, Table 3, respectively, showing the estimated regression parameter and the value of the corresponding t-statistic for each independent variable.

In a MMNL model, the estimated parameters alone are not sufficient to explain the actual effect of a variable on injury severity. It is thus important to consider the marginal effects given by the pseudo-elasticity in addition to the parameter values [42]. The average direct pseudo-elasticity for the model parameters of the nighttime and daytime models and each severity level are listed in Table 4 and Table 5, respectively. The selected independent variables can be classified as descriptors related to the driver, crash information, roadway geometry, environment or work zone information. For better understanding, the modeling results and discussion are presented under the same sections.

**Table 4:** Average Direct Pseudo-elasticity effects for nighttime model.

Variable	Elasticity %		
	PDO	Injury	Fatality
Young	96.63	-15.32	23.31
Old	6.59	0.05	-1.33
Male	-142.23	-7.94	47.2
Curve	-9.84	-4.88	14.85
U-turn	-30.96	15.25	-35.11
Rural	-72.58	0.28	12.52
Angle	13.93	-2.31	-14.04
Sideswipe	87.58	-16.79	28.9
Heavy	-49.17	-8.06	30.52
Passenger	-327.67	0.42	58.73
Multi-vehicle	82.98	22.17	-74.44
Winter	-26.43	-1.3	8.31
Rain	-28.54	8.59	17.75
Concrete	-11.56	8.22	-19.87
Asphalt	-18.02	2.07	-2.24
Milling	31.63	3.31	-14.63
U-turn	-30.96	15.25	-35.11
N. lane closures	-21.78	-11.61	35.02
Speeding	-76.87	1.47	10.12

**Table 5:** Average Direct Pseudo-elasticity effects for daytime model.

Variable	Elasticity %		
	PDO	Injury	Fatality
Young	-2.96	-1.12	133.08
Old	-8.42	1.39	9.55
Male	50.51	-8.36	-53.7
Curve	16.86	-3.87	69.04
Rural	-107.0	18.8	26.81
Sideswipe	-7.9	2.38	-78.33
Pedestrian	-10.67	1.82	6.54
Heavy	-12.26	1.48	57.1
passenger	-205.5	29.92	549.44
Multi-vehicle	210.81	-34.1	-287.94
weekday	22.25	-2.08	-153.46
Rain	2.61	0.35	-66.2
Fog	29.76	-4.49	-67.01
Concrete	-17.84	4.39	-96.82
Asphalt	-24.69	4.82	-32.98
Milling	6.68	-0.73	-37.61
Remove	9.76	-0.86	-71.76
N. lane closures	38.68	-6.15	-61.79

#### Driver characteristics

Crashes during either daytime or nighttime are often associated with the at-fault driver being in a younger age group (less than 35 years old) or older-aged (over 50 years of age). For instance, the pseudo elasticity for the older age group indicates that there is a 9.6% increase in the likelihood of fatality for older drivers in daytime crashes, whereas there is a decrease of 1.3% during nighttime crashes.

The opposite effects of the old age factor on injury severity in nighttime and daytime crashes show a statistical difference between the two models. For young drivers, the probability of being involved in severe work zone crashes increases by 133% and 23.3% during daytime and nighttime, respectively. In the older age group, slower perception and longer response times may account for the higher severity levels [43], which have been associated with weak physical condition and excessive mental stress [44]. In the younger age group, the effect may be explained by lack of driving experience and a tendency of driving at high speed during nighttime [45]. Special attention should therefore be paid to these age groups.

With respect to gender, the results show the positive pseudo elasticity (47.2%) of male drivers indicates a higher probability of being involved in fatal nighttime work zone crashes than female drivers. On the other hand, male drivers are less likely to be involved in fatal crashes (53.7%) during the daytime. This may be representative because male drivers tend to take more risks, drive over the speed limit and drive more aggressively. This is consistent with the findings of previous studies [6,46]. However, other studies found that female drivers have a higher risk of suffering injuries from work zone crashes [47].

#### Roadway characteristics

Work zone crashes have been shown to be associated with road characteristics. The positive pseudo-elasticity of both models implies that work zone crashes occurring on curved sections have a tendency towards having high injury severity. On curved sections the probability of suffering fatal injuries increases by 69.0% and 14.8% during daytime and nighttime, respectively. Notably, the increase is more significant during the daytime. This is also consistent with the findings of previous researches [48,49]. Other studies present the observation that fatal work zone crashes are less influenced by horizontal and vertical curves compared to non-work zones collisions [50,51]. In a study by Katta, curved road geometries were not found to be a significant variable [52].

#### Crash characteristics

In work zone crashes, the types of vehicle involved are strongly associated with injury levels. The probability of injury increases (1.5%) during daytime and decreases (8%) during nighttime when a heavy vehicle is involved. The severity levels, however, increases significantly with the probability of fatality increasing to 57.1% and 30.5% at daytime and nighttime, respectively. It is well known that incidents involving heavy vehicles in work zones statistically lead to multiple vehicle crashes as well as fatalities due to the reduced braking capability of such vehicles. For passenger cars, the risk of a fatal crash in work zones increases by 549.4% and 58.7%, during daytime and nighttime respectively. This result reveals that the probability of passenger car being involved in fatal crash increasing more significantly during daytime than nighttime (549.4% compared to 58.7%). The impact of heavy duty and passenger types of vehicle on work zone crash injury severity has been established in several studies [6,50,53–55]. In particular in Egypt, large volume and excessive weight of trucks serve as an explanation for this dependence, due to that 96% of goods transport in Egypt is by road [1].

When multiple vehicles are involved in crashes at night, the injury severity tends to increase (22.2%), whereas it decreases during the day (34.1%). This may be because when more vehicles are involved in nighttime incidents, the multiple impacts worsen the situation due to higher speeds and lower visibility. This is consistent with some earlier research [56,57], but in contrast with other findings [58]. In multiple vehicle incidents, sideswipe crashes between vehicles moving in the same direction increase the fatality risk by 28.9% at night but decrease by 78.3% during the day. This effect can be explained by the narrowed traffic lane width, influencing the interaction between vehicles.

Angle and U-turn crashes are associated with injury severity of nighttime crashes, but has no significance during the day. The finding indicates that drivers involved in angled collisions during U-turn maneuvers tend to decrease the probability of causing fatality by 14% since such maneuvers usually are performed at low speeds. The U-turn is considered one of the most dangerous maneuvers a driver can make and contributes directly to angle crashes at nighttime. This is in agreement with other studies [50].

The area type in which work zone incidents occur is also important. In rural areas, the probability of fatal crashes tends to increase by 26.8% and 12.5% during the daytime and nighttime, respectively, compared to urban areas. It may be because of the higher speed limits in rural areas. The presence of pedestrians or construction workers are found to be a significant factor only in the daytime model. Collisions between vehicle and pedestrians are then likely to increase the fatality rate by 6.5%. This might be due to insufficient protection of construction workers [23].

#### Environmental characteristics

Weather conditions are significant risk factors in work zone crashes. The results indicate that during rainy weather, fatal collisions are less likely by 66.2% during daytime but occur at an increased rate of 17.8% during nighttime. The main cause of the increase in severity level is that a wet road surface could make it slippery, thus making the breaking distance longer compared to dry surfaces. During foggy weather the probability of being involved in a fatal crash tends to decrease by 67.0% during the day. This indicates that daytime crashes during rainy and foggy weather are less likely to result in fatalities. An explanation for this is that more careful driving during adverse weather and heavy traffic conditions during daytime leads to lower speed. Higher fatality rates were found to be significant during the winter period at night, but not so in the daytime model. This is likely because rainy weather is common during the Egyptian winter season. Osman et al. also found that rainy weather is significantly associated with crash severity at work zones [59]. However, research by Harb et al. suggested that rain tends to decrease the injury severity [51]. Possibly more effective traffic safety measures can be developed for specific adverse weather conditions.

The factor ‘Weekday’ was found to be associated with fatal crash injuries during the daytime, decreasing by 153.4%, but insignificant during nighttime. This is likely due to the fact that drivers who commute daily during weekdays have a better visual impression and longer time to discern work zones and to react accordingly–they have more time to slow down or take other actions to reduce the impact risk. These findings are consistent with previous research [23,24,60] where work zone crashes were found to be more likely on weekends.

#### Work zone information

Detailed advance information about work zones configuration like the speed limit, construction type and the number of lane closures are also found to exhibit a significant relationship with the severity of work zone crashes. In highway construction, some factors are found to be significant in both models, but still exhibit considerable differences. For example, during asphalt pavement construction conditions, the probability of fatalities during the day decreases more significantly than during the night (33.0% compared to 2.2%). By comparing the nighttime surface construction works, the probability of fatality is decreasing more significantly for concrete than for asphalt pavement construction conditions (19.8% compared to 2.2%). Other variables such as milling and concrete pavement construction show a similar pattern.

The coefficients for the number of lane closures show opposite signs, where the closure of many lanes is associated with an increased rate of fatalities by 35.0% during the nighttime. At the same time, the fatality risk decreases by 61.8% during the day, indicating a more careful driving due to heavy traffic conditions. A likely reason for the higher risk at night is forced merging of traffic, which could be attempted late due to poor visibility and high speed, thereby leading to increased severity. In particular heavy vehicles can be difficult to maneuver in work zones if changing lanes is commenced late. Previous studies on traffic safety at work zones have concluded that the number of lane closures has a significant relationship with work zone crashes [61–63].

Exceeding the speed limits is a strong influencing factor on nighttime work zone crashes. The positive pseudo-elasticity of the variable indicates that the injury severity level increases the more the speed limit is exceeded. A reasonable explanation is that low visibility in combination with higher speeds made possible by lighter traffic greatly increases the fatality rate. This is in agreement with several previous studies [53,60,64]. The strong correlation between speed and crash severity was also found by Meng et al., reporting a 62% decrease in fatality rate and a 44% reduction in injury rate when the mean travel speed is slowed down by 20%. This shows that speed reduction in work zones is crucial in bringing down crash severity levels [65]. The models indicate a similar situation for Egyptian roads, further exacerbated by the fact that not all highways have speed control devices, especially not in work zones.

The summary results of the current study clearly indicate that there is difference between the influence of a variety of variables on the injury severities resulting from day and nighttime work zone crashes. For example, 4 variables are significant only in the daytime model. Similarly, 4 different variables are significant only in the nighttime model. Finally, 14 variables are found significant in both day and nighttime models.

## Model specification and transferability tests

### Model specification test

Although substantial differences between day and nighttime crash models specifications are identified, their model similarity and generality need to be statistically examined to justify the necessity of developing and estimating two mixed logit models for injury severity analyses in day and nighttime crashes, respectively. The likelihood ratio test comparing the fixed-and random-parameter models is performed to test the null hypothesis that the MNL model is statistically equivalent to the MMNL model. The chi-square statistic is

$$X^{2}=-2[L_{MNL}(\beta )-L_{MMNL}(\beta )]$$ (7)

where *L*_*MNL*_(*β*) is the log-likelihood at convergence of the MNL model and *L*_*MMNL*_(*β*) the log-likelihood at convergence of the MMNL model. The chi-square statistic for the likelihood ratio test with two degrees of freedom gives a confidence limit based on one-tailed *p*-value greater than 99.99% (χ^2^ = 95.2) for the daytime model and (χ^2^ = 110.3) for the nighttime model, indicating that the MMNL model is statistically superior to the MNL model.

### Statistical tests of temporal transferability

If separate models are estimated using two separated dataset with the same variables, an important consideration is to test for transferability of the models. To do this, the likelihood ratio test comparing the combined model (daytime and nighttime crashes) and two separate models for nighttime and daytime was performed to test the transferability of coefficients in nighttime and daytime separately. The chi-square statistic is

$$X^{2}=-2[L_{Nall}(\beta )-L_{Nnight}(\beta^{night})-L_{Nday}(\beta^{day})]$$ (8)

Where *L*_*Nall*_(*β*) is the log-likelihood at convergence of the model for the complete data set, and *L*_*Nnight*_(*β*^*night*^) and *L*_*Nday*_(*β*^*day*^) are the log-likelihood at convergence of the model for the nighttime data subset, and the daytime data subset, respectively. The chi-square statistic for the likelihood ratio test gives a confidence limit based on one-tailed *p*-value greater than 99.99% (χ ^2^ = 151.03) with number of degrees of freedom equal to the sum of the number of the estimated parameters in the nighttime and daytime models minus the number of the parameters estimated in the data models. This indicates a significant difference of severity between nighttime and daytime crashes, which also justifies modeling nighttime and daytime crashes separately.

## Conclusions

A MMNL regression model is fitted to work zone crash data to determine significantly influencing factors on injury severity following day-and nighttime collisions. The analysis is based on data consisting of 1,380 incidents in work zones in Egypt between the years 2010 and 2016. A large collection of different risk factors including driver attributes, crash characteristics, roadway and environmental conditions, and work zone configurations were included in the mixed logit models. The results of the analyses provide some interesting findings on injury severity, which are summarized in the following.

1. The likelihood ratio test indicated that estimating two separate models for day and night incidents is statistically justifiable.
2. The results showed substantial differences in significant factors between the nighttime and daytime models. Some factors, for example day of the week, foggy weather, pedestrian crash and removed asphalt surface are only significant in the daytime model, whereas the factors representing excessive speeding, winter season, angle crashes and U-turn maneuvers are only significant in the nighttime model.
3. Even if some variables were found to be significant in both nighttime and daytime models, there is considerable difference of the marginal effects on these two models. Some of them showed opposite effects for nighttime and daytime crashes. Other variables show a large difference in magnitudes even when having the same trend.

Crashes in rainy conditions during nighttime tend to increase the fatality rate, whereas it decreases during the day. Night incident injury severity is also found to increase with speeding, particularly for male drivers. Passenger vehicles are more likely to be involved in fatal crashes during the day than during the night. Furthermore, fatalities are more likely during the day whenever pedestrians and workers are involved.

Interesting results were found for the younger and older age group of drivers. The model showed that older drivers were associated with a decreased probability of fatal crashes at night, but an increased probability during the daytime. Contrary to this, young drivers have a higher risk of fatal crashes during the day than at night. The study shows that during nighttime, passenger and heavy vehicles driving at high speed in work zones with lane closures and making sudden maneuvers to change lanes, are at high risk for being involved in fatal crashes.

The findings from this analysis can be expected to facilitate transport agencies in the development of efficient measures to ensure safety at work zones, mitigate risk factors and increase the traffic safety on Egypt’s highways.

Since most crashes can be attributed to human error, advanced knowledge of the work zone conditions allows drivers to prepare for hazardous situations more adequately. For this reason, it is recommended to develop an effective educational program for the general public on precautions that should be taken when driving through work zones.

Furthermore, ITS technologies, such as variable speed limit (VSL) and dynamic message signs (DMS) at an appropriate distance ahead of the work zone, can provide efficient means to provide drivers with updated information. Providing dynamic information can mitigate the risks of adverse design, human factors, and roadway conditions, as well as balancing the traffic volumes between lanes, thus reducing lane-changing maneuvers.

Other more conventional traffic engineering solutions include increasing the upstream distance from the work zone where illumination or fluorescent devices, cones and barrels are placed, as well as enhancing visibility and reducing the speed limit.

The outcome of the present analysis is limited by available data, which may affect results and their interpretations. An example hereof is that police reports do not include detailed crash location within the work zone (whether the incident occurred in the advance warning area, transition area, activity area or termination area). A limitation of available data in this study is that some potentially crucial information (such as seat belt usage, alcohol levels, etc.) have not been considered since the data are missing from the database. It is therefore advisable that more detailed information be collected and entered into the database so that it could be used for further model calibration and a more detailed analysis of risk factors in different crash scenarios.

In terms of future work, there is a need to assess the potential spatial correlation among adjacent work zones, and the obvious interactions among work zone–related factors to provide important new insights into day and nighttime crashes occurrence.
